# Supplementary material for: Are carbon emissions trading and green financial instruments synergistic? -Comprehensive quantitative research based on content analysis
Source: PLoS One. 2024 Mar 7;19(3):e0298601. doi: 10.1371/journal.pone.0298601 (PMC10919601; doi:10.1371/journal.pone.0298601)
Supplement: S1 Data — (ZIP) [file pone.0298601.s001.zip › Data/地级市Z_F工作报告.zip!/║╙─╧/╨┼╤⌠╩╨/└┤╫╘╣1⁄2╓┌║┼í╛┬φ┐╦╩2╛▌═°í┐_2019.docx]

各位代表：

　　现在，我代表市人民政府，向大会报告工作，请予审议，并请各位政协委员和其他列席同志提出意见。

　　一、2018年工作回顾

　　过去的一年，在省委、省政府和市委的正确领导下，全市上下以习近平新时代中国特色社会主义思想为指导，认真贯彻落实党的十九大和习近平总书记视察指导河南时的重要讲话精神，深入践行新发展理念，按照高质量发展要求，齐心协力，攻坚克难，经济社会发展呈现总体平稳、稳中有进的良好态势。全市生产总值完成2387.8亿元，增长8.3%，增速居全省第4位。固定资产投资增长9.7%，社会消费品零售总额增长10.8%，规模以上工业企业增加值增长7.6%，均高于全省平均水平。一般公共预算收入完成110.74亿元、增长10.2%，其中税收完成77.6亿元、增长15.8%；居民人均可支配收入增长9.6%，居全省第3位。

　　（一）三大攻坚战成效明显。有效防范重大风险。政府性债务管理不断加强，非法集资连续两年保持低发案率，银行业不良贷款风险总体可控，稳妥化解“6.11”涉军群体聚集活动。打好打赢脱贫攻坚战。扎实推进“四场硬仗”“六大行动”“四大工程”，全年实现脱贫18.8万人，完成易地扶贫搬迁3997人，固始县、光山县、潢川县、商城县、罗山县、息县成功脱贫摘帽，在全省脱贫攻坚成效考核中居第5位。新增“多彩田园”产业扶贫示范基地1178个，解决611个行政村安全饮水问题，改造农村危房7.4万户，教育资助政策全面落实，“3+2+N”医疗保障体系基本形成，所有贫困村通硬化路、实现广播电视户户通和综合性文化服务中心全覆盖，全国贫困重度残疾人家庭无障碍改造现场会，全省旅游扶贫推进会、职业教育助力脱贫攻坚现场会、交通运输脱贫攻坚暨“四好农村路”建设现场会均在我市召开，一元坐公交的“潢川样本”被全省推广，新县脱贫摘帽的成功实践被央视等主流媒体连续报道。大力推进污染防治。开展“三大专项行动”，成立“四个专班”，落实“六控”措施，积极创建国家空气质量二类标准城市。全面落实河湖长制，中心城区黑臭水体水质达标51处、完成率82%，河流断面水质保持稳定，城市集中式饮用水水源水质达标率100%，成功争取全国首批黑臭水体治理示范城市。树牢政治意识，提升政治站位，坚决推进河道采砂专项治理，实现淮河干流全面禁采，支流依法科学规范有序开采。推进国家森林城市创建，在全省率先开展大规模国土绿化提速行动，完成新造林36.79万亩、森林抚育67.44万亩，分别增长59.6%和33.7%。

　　（二）项目支撑显著增强。深化“项目建设年”活动，448个省市重点项目完成投资667.8亿元，基础设施领域投资增长18%。淮滨至信阳高速公路息县至邢集段完成工程量的60%以上，淮河淮滨至息县航运工程淮滨段完成工程量的70%以上。出山店水库移民搬迁、库底清理全部完成，防护工程建设基本完成，顺利通过水利部和省政府阶段性验收。明港机场正式通航，旅客吞吐量已突破30万人次。安阳至罗山高速公路上蔡至罗山段、许昌至信阳高速公路信阳段纳入省高速公路“双千工程”，G107、G312绕市区段一级公路新建工程基本具备开工条件。大别山革命老区引淮供水灌溉、洪汝河治理（淮滨段）、五岳抽水蓄能电站、袁湾水库等工程前期工作基本完成。加大项目谋划争取力度，沿大别山高速公路、淮滨至内乡高速公路豫皖省界至淮滨段、濮阳至阳新高速公路商城至豫皖省界段、安阳至罗山高速公路罗山至豫鄂省界段纳入省“十三五”高速公路网规划，京九高铁阜阳经潢川至黄冈段有望纳入中铁总公司“十四五”建设计划，合康高铁襄阳经随州、信阳至合肥段规划研究报告编制完成，淮河航运工程纳入省重大战略，淮河淮滨至息县航运工程息县段纳入交通运输部“十三五”中期调整方案，张湾水库、晏河水库、出山店水库灌区等工程前期工作取得阶段性进展，潢川机场纳入国家“十三五”规划中期调整方案。抢抓淮河生态经济带上升国家战略的重大机遇，专题谋划8类工程2461个项目，信阳发展后劲进一步增强。

　　（三）产业转型步伐加快。工业经济持续增长。工业投资增长16.6%，居全省第2位；规模以上工业企业主营业务收入增长16.4%，居全省第3位。推进食品、建材、纺织服装、装备制造4个传统支柱产业脱胎换骨式改造，实施“三大改造”项目249个，创建国家级绿色工厂1家、省级智能工厂2家、省级智能车间6家。大力培育高技术、高成长、高利税企业，信阳高新区创建国家级高新区通过国家部委审核，新认定高新技术企业32家，登记入库国家科技型中小企业181家。特色农业不断壮大。新建高标准粮田57.3万亩，优质水稻面积稳定在600万亩以上，新发展弱筋小麦61.1万亩、稻渔综合种养32.16万亩、再生稻5.4万亩、油茶3.9万亩。“信阳毛尖”入选首届中国农民丰收节全国100个农产品品牌，“南湾鱼”获中国驰名商标。新增省级农业产业化联合体19个、农业标准化生产示范基地6个。信阳农业科技园获批国家农业科技园区，潢川县获评全国渔业健康养殖示范县。现代服务业融合发展。全市服务业增加值增长12.6%，居全省首位。推进茶旅深度融合，打造环南湾湖茶旅融合精品线路，建成5个茶旅融合示范点和“十大茶坊”，认定信阳菜品牌示范店60家，全年实现旅游收入313.3亿元、增长38.6%。编制“两湖”区域发展规划，启动中国健康（信阳）康养示范基地建设。实施34个物流转型发展重点项目，全市冷链物流总库容近60万立方米，省级电子商务进农村综合示范县实现全覆盖。

　　（四）改革开放不断深化。深化“放管服”改革，实施“一网通办”前提下“最多跑一次”改革，实现“一网通办”事项962项，企业开办时间压缩至4.5个工作日，全市市场主体达27.5万户、增长14.2%。114户企业改革处置任务圆满完成，被评为全省国企改革攻坚工作先进市。深入推进土地利用综合改革，节余指标交易数量和收入居全省首位。农村“三权分置”改革进展顺利。政府机构改革基本完成。全面推进开放招商，大力开展产业链招商、园区招商、协会招商、委托招商，持续实施回归工程，成功举办首届信商大会和国际纺织服装加工贸易峰会暨2018河南服装大会，累计引进省外资金项目273个，实际到位省外资金281.7亿元，13.4万人返乡创办各类市场主体7.9 万个、带动就业89.5万人。外贸实绩企业达141家，外贸进出口总额增长27.5%。不断增强创新活力，建成OVU创客星·信阳高新区智慧信息创新创业示范基地，新增国家级星创天地2家，新建省级院士工作站3家、工程技术研究中心9家，获省科技进步奖6项，引进科技成果13项，省级新型研发机构实现零突破。依托中国·河南招才引智创新发展大会，签约引进人才项目81个，成功获批全省首家留学人员创业园。

　　（五）城乡发展更趋协调。以百城建设提质工程为载体，推进城市内涵式发展。坚持规划先行，完成浉河三期、信阳东站、火车站北广场、海营湿地等重点区域城市设计，基本实现中心城区控规全覆盖。开展城市“双修”，中心城区30个重点项目建成2个、在建24个、即将开工4个，火车站北站房建成投用，跨浉河三座桥、羊山森林植物园、四一路铁路立交桥改扩建工程按时序推进，新十八大街贯通工程开工建设、北环路以北8.1公里建成通车。完成园林绿化项目17个、生态水系治理项目5个，新建改建城市公厕173座，新增新能源公交车300台。明阳新能源装备产业园、谷麦光电中部半导体等产城融合项目落地建设。巩固国家卫生城市创建成果，启动新一轮全国文明城市创建，出台《信阳文明20条》，以李道洲、李芳为代表的信阳好人享誉全国。大力实施乡村振兴战略，制定发展全域旅游助力乡村振兴三年攻坚计划，推广新县经验，实施乡村旅游发展、旅游品牌创建、旅游商品开发等13项攻坚行动，创建省级乡村旅游特色村18个，全市依托全域旅游实现脱贫1.4万人、增长20%。实施农村人居环境整治三年行动，完成户厕改造12.3万户，新增人居环境达标村240个、示范村168个，淮滨县、光山县、浉河区农村垃圾治理通过省级达标验收。创建全国生态文化村2个、省级以上传统村落29个，光山县帅洼村入选2018年中国美丽休闲乡村，新县被授予国家生态文明建设示范县。

　　（六）民生福祉持续增进。财政支出近八成投向民生领域。城镇新增就业10.57万人，3.21万人实现再就业。省重点民生实事落地见效，被省政府通报表彰；市十件实事已完成8项，剩余2项今年9月底前全部完成。加快发展社会事业，新建改扩建公立幼儿园88所、农村寄宿制学校64所，“全面改薄”圆满收官，义务教育超大班率控制在2.34％，商城县、淮滨县、息县通过国家义务教育发展基本均衡县评估验收，信阳“五维督查”助推特岗教师计划落实入选国务院2018年全国政府系统督查实践精品案例。大别山高中主体工程完工，大别山艺术职业学院纳入河南省高校设置“十三五”规划，信阳航空职业学院挂牌招生。“三医”联动改革和分级诊疗建设取得新突破，全省县域综合医改现场会在息县召开。大力发展群众体育和竞技体育，实施文化惠民工程，6家公共图书馆获评国家一级馆，新增国家级非物质文化遗产代表性项目传承人3人，商城县上榜“中国民间文化艺术之乡”。兜住民生底线，城乡居民最低生活保障月人均财政补助标准分别提高到262元和154元，城乡特困人员基本生活标准分别提高到每人每年7332元和4486元。完成贫困重度残疾人家庭无障碍改造2.2万户，11.2万名残疾人获“两项补贴”。安置房年度建成率177%，分配公租房6201套。扫黑除恶专项斗争深入开展，安全生产、食品药品安全、信访稳定、社会治安综合治理形势总体平稳。

　　各位代表！一年来，我们树牢“四个意识”，坚定“四个自信”，做到“两个维护”，确保党中央国务院各项决策部署落地落实。加强法治政府建设，推进依法行政，自觉接受人大法律监督和政协民主监督，办理人大代表建议189件、政协委员提案434件，满意率分别达99.5%和95%。切实履行全面从严治党主体责任，严格执行中央八项规定精神，强化审计监督，驰而不息纠正“四风”，扎实开展巡视反馈问题整改，党风廉政建设不断加强，政府治理能力和服务水平持续提升。

加强国防教育、国防动员、人民防空、退役军人服务管理等工作，支持军队改革发展，推进军民融合发展，驻信解放军、武警部队、预备役人员、民兵为地方发展作出了新贡献，双拥工作持续深化。工会、共青团、妇联、红十字会、法学会等群团组织在经济社会发展中发挥了不可替代的作用，民族宗教、外事侨务、对台工作全面加强，统计、档案、气象、地方志、社科研究、防震减灾、机关事务管理、援疆等工作取得了新的成绩。

　　各位代表！过去的一年，外部环境复杂严峻、转型阵痛压力加大、风险挑战前所未有，能够取得这样的成绩，是习近平新时代中国特色社会主义思想科学指引的结果，是省委、省政府和市委坚强领导的结果，是市人大、市政协监督支持的结果，是全市人民一起奔跑、同心追梦的结果。在此我代表市政府，向全市广大工人、农民、知识分子，向驻信部队指战员、武警官兵、公安民警、消防救援队伍，向中直、省直机关和金融机构驻信单位，向各民主党派，向全市新闻工作者，向在我市创业的境内外投资者、建设者及社会各界人士，致以崇高的敬意和衷心的感谢！

　　我们也清醒地认识到，全市经济社会发展还存在一些困难和问题，主要表现在：经济下行压力加大，实体经济困难较多，市场需求增速放缓，营商环境与市场主体期待还有较大差距；转型发展动力不足，新兴产业规模偏小，新技术、新业态、新产品、新模式依然较少；民生领域还有不少堵点痛点，群众在教育、医疗、养老、住房、出行等方面还有不满意的地方。同时，正反向激励不够，一些公务人员在探索发展路径、推动工作落实上，用心不专、用力不足、用劲不实。对此，我们一定高度重视、认真解决。

　　二、2019年工作安排

　　2019年是新中国成立70周年，是全面建成小康社会、实现第一个百年奋斗目标的关键之年，做好今年工作意义重大。

今年政府工作的总体要求是：以习近平新时代中国特色社会主义思想为指导，全面贯彻党的十九大和十九届二中、三中全会精神，深入贯彻落实习近平总书记视察指导河南和参加十三届全国人大二次会议河南代表团审议时的重要讲话精神，统筹推进“五位一体”总体布局，协调推进“四个全面”战略布局，坚持稳中求进工作总基调，坚持新发展理念，坚持推动高质量发展，坚持以供给侧结构性改革为主线，坚持深化市场化改革、扩大高水平开放，贯彻“巩固、增强、提升、畅通”八字方针，统筹推进稳增长、促改革、调结构、惠民生、防风险、保稳定工作，落实“六稳”工作部署，扎实开展“干部作风提升年”活动，着力打造“一市一区三枢纽”、加快建设“五个信阳”，为全面建成小康社会收官打下决定性基础，在造福信阳、添彩中原的征程中迈出更大步伐。

　　今年经济社会发展的主要目标是：地区生产总值增长8%—8.5%，一般公共预算收入增长8%以上，税收占一般公共预算收入的比重不低于70%，固定资产投资增长9%以上，社会消费品零售总额增长10%以上，居民消费价格涨幅3%左右，经济结构进一步优化，质量效益明显提升。五大发展理念落地见效，研究与试验发展经费支出增长20%以上，高技术产业、战略性新兴产业增加值均增长10%以上；常住人口城镇化率提高1.5个百分点以上，九年义务教育巩固率达到95%，医疗卫生、文化、体育等社会事业全面进步；单位生产总值能耗下降3%以上，主要污染物排放量完成省下达目标；进出口保持平稳增长，营商环境在全省评价排序中力争取得较好位次；居民人均可支配收入增长8.5%，城镇新增就业9万人以上，城镇登记失业率控制在4%以内。

　　为实现上述目标，今年以来我们保持定力，增强耐力，勇于攻坚克难。一季度，全市生产总值达539.75亿元，同比增长8.6%，增速居全省第3位；规模以上工业企业增加值同比增长9.6%，增速居全省第1位；一般公共预算收入同比增长11.5%，社会消费品零售总额同比增长10.5%，固定资产投资同比增长8.3%，顺利实现“开门红”。我们将坚持把发展扭住、把重点抓住、把风险管住、把民生兜住，切实抓好以下六个方面工作，确保完成全年各项目标任务。

　　（一）深入实施乡村振兴战略，开创“三农”工作新局面。认真学习贯彻习近平总书记在河南团的重要讲话和省委十届九次全会精神，坚持农业农村优先发展，探索乡村振兴的信阳路径。

　　发展壮大现代农业。扛稳粮食安全重任，落实藏粮于地、藏粮于技，坚守耕地红线，新建高标准农田54万亩，粮食产能稳定在110亿斤以上。大力推进农业供给侧结构性改革，聚焦“三链同构”，持续实施“四调”，优质弱筋小麦、稻渔共作、再生稻面积分别发展到200万亩、60万亩、20万亩，新增油茶面积5.5万亩；加快粮食产业“产购储加销”一体化发展，延长“稻虾共作”产业链，推进花卉苗木产业由种植向花卉游延伸。完善农产品原产地可追溯制度和质量标识制度，培育油茶和中药材品牌，叫响“信阳毛尖”“信阳菜”等特色农产品品牌。发展多种形式适度规模经营，突出抓好家庭农场和农民合作社两类农业经营主体发展，推进小农户和现代农业的有机衔接，形成“龙头企业+合作社+基地+农户”发展体系。大力发展“互联网+”，推进电子商务县乡村三级物流体系建设，鼓励企业将分支机构延伸到乡村，打通工业品下乡、农产品进城双向流通渠道。

　　大力发展全域旅游。积极创建大别山北麓全域旅游示范区，编制全域旅游发展规划，加快景区创建，着力发展绿色生态游、红色研学游、乡村游和民宿游，全年实现接待游客4800万人次以上，旅游综合收入400亿元以上，力争新县国家全域旅游示范区创建成功。实施茶园增色添彩工程，以环南湾湖茶旅融合区域为示范，统筹推进浉河区、鸡公山管理区、罗山县、新县、商城县、潢川县、固始县、光山县等茶旅综合体建设，加快建设平桥区陆庙茶山居项目，打造一批茶旅融合精品线路。实施“品信阳菜、喝毛尖茶、看淮上山水、住豫南民宿”工程，鼓励茶企、茶人开办茶馆，营造全民“知茶、爱茶、饮茶”的氛围。加强信阳菜挖掘与推广，加快信阳菜工程研发中心、中央厨房、配送中心建设，争创河南传统餐饮历史文化名城，打造河南茶筵之乡。推进浉河区四望山新四军第五师师部旧址、商城金刚台红军洞群、新县箭厂河革命旧址和新县将军故里4个全国红色旅游经典景区建设，举办红二十五军长征出发85周年纪念大会暨首届中国乡村振兴何家冲论坛；推动大别山干部学院与市委党校、县区党校资源整合，在全市红色旅游景点布局一批现场教学点；启动黄湖农场“五七干校”陈列馆建设，打造全国青少年培训基地。放大全球文旅创作者大会效应，办好第五届全国民宿大会，每个县区培育特色生态旅游示范乡镇2个以上、乡村旅游示范村2个以上、规模以上特色民宿3家以上，把信阳变成一座旅游“网红”城市。推进旅游业与文化、健康、养老、体育等产业融合发展，以“两湖”区域为重点，建设国家生命健康产业创新示范区，积极争取国家级康养产业试点市。持续推进旅游高速路、干线公路与景区公路互联互通，提高乡村旅游道路等级，打通3A级以上景区和重点旅游村“最后一公里”。

　　扎实推进乡村建设。坚持规划引领，统筹土地利用、产业发展、居民布点、人居环境、生态保护和历史文化传承，注重乡村振兴规划与乡村总规、旅游规划、土地空间规划及其他规划融合衔接，完成县域乡村建设规划和50%以上行政村实用性村庄规划编制修编。突出抓好农村人居环境改善，学习借鉴浙江“千万工程”经验，实施“百千工程”，重点打造100个示范村、1000个达标村；实施“三清一改”村庄清洁行动，所有县区农村生活垃圾治理通过省级达标验收，全市90%以上行政村生活垃圾得到有效治理，县域农村生活污水治理率达到23%，秸秆综合利用率达到88%，畜禽粪污资源化利用率达到78%；打赢“厕所革命”攻坚战，完成农村改厕20万户；开展“四美乡村”“五美庭院”“美丽小镇”创建活动，打造50个美丽乡村，建设10个特色文化小镇，提升村容村貌、户容户貌、人容人貌。加快补齐农村交通运输、安全饮水、宽带网络等基础设施短板，推进驻马店挚亭至信阳春申500千伏线路工程建设。深化拓展“四议两公开”工作法，发挥好新乡贤作用，激发群众内生动力，健全自治、法治、德治相结合的乡村治理体系。聚焦乡风文明，推进移风易俗，培育文明乡风、良好家风、淳朴民风；传承弘扬豫风楚韵的优秀传统，让有形的乡村文化留得住、活态的乡村文化传下去，让乡村更富魅力、更具吸引力。

　　持续深化农村改革。深化国家农村改革试验区建设，统筹推进10项改革任务，加快农村土地征收、集体经营性建设用地入市、宅基地制度“三块地”改革，探索农村集体经济新的实现形式和运行机制，推动资源变资产、资金变股金、农民变股东。充分发挥本地“田专家”“土秀才”作用，大力培育新型职业农民和专业技术人才，搭建平台吸引优秀人才返乡创业。

　　决战决胜脱贫攻坚。坚持以脱贫攻坚统揽经济社会发展全局，把脱贫攻坚作为乡村振兴的优先任务，目标不变、靶心不散、频道不换，确保5.2万贫困人口脱贫，淮滨县高质量脱贫摘帽。严格“两不愁三保障”标准，紧盯薄弱环节，持续加大危房改造力度，确保4类重点对象危房改造全部清零；落实好建档立卡贫困家庭学生教育保障和资助制度，确保贫困家庭子女顺利完成义务教育；巩固“3+2+N”医疗保障体系，确保建档立卡贫困人口全部纳入医疗救助保障范围，实现贫困户慢性病卡办理和家庭医生签约服务全覆盖。提升产业扶贫“多彩田园”示范工程质效，促进贫困群众稳定增收。坚持问题导向，按照“四个查一查”要求，查漏补缺，全面整改；统筹解决非贫困村发展、基础设施和公共服务不均衡等问题。坚持“四个不摘”，保持脱贫攻坚政策连续性、稳定性，重点关注因病致贫户、无劳动能力户，突出抓好“六类人”保障兜底。脱贫攻坚战进入决胜的关键阶段，务必一鼓作气、顽强作战，不获全胜决不收兵。

　　（二）全力抓好项目建设，积蓄高质量发展后劲。坚持以项目为抓手，扩大总量、优化存量、提升增量，夯实高质量发展基础。

　　积极谋划项目。围绕淮河生态经济带发展规划、国家基础设施领域补短板等政策，动态策划包装项目，市本级安排经费9000万元支持项目前期工作，动态储备项目库保持在1万亿元以上，年实际争取上级资金25亿元。推动大别山北麓旅游高速公路纳入省交通“十三五”项目库，完成沿大别山高速公路鸡公山至商城段工可及初步设计批复、达到专项债申报条件，完成濮阳至阳新高速公路商城至豫皖省界段、安阳至罗山高速公路罗山至豫鄂省界段工可批复，完成信阳至随州高速公路工可编制。推进京九高铁阜阳经潢川至黄冈段前期工作、争取“十三五”开工建设，积极争取合康高铁襄阳经随州、信阳至合肥段纳入国家铁路网规划，联合沿淮城市科学谋划沿淮高铁。加快推进淮河航运工程，建成淮滨至息县航运工程淮滨段，完成淮滨至息县航运工程息县段前期工作，争取淮河干流息县至长台关段纳入交通运输部“十四五”规划。加快推进淮滨临港经济区“一港六区一廊道”建设，打通到沿淮知名港口的航运专线。完成出山店水库下闸蓄水，开展库区生态环境和美好移民村建设。启动明港机场扩能改造，加快潢川机场前期工作。

　　狠抓项目建设。落实“7819”扩大有效投资专项行动，把重大项目落地开工作为“一号工程”，以20个重大项目为引领，确保总投资6389.77亿元的1709个重大项目完成年度投资1744.13亿元。1、开工建设（5月底前）五岳抽水蓄能电站；2、启动（8月底前）国家干细胞健康工程产业示范区建设；3、开工建设G312绕市区段一级公路新建工程；4、开工建设（8月底前）大别山艺术职业学院一期工程；5、开工建设（9月底前）河南省大别山革命老区引淮供水灌溉工程；6、开工建设（9月底前）信阳市中心城区静脉产业园（生活垃圾焚烧发电项目一期）；7、开工建设中心城区调水及生态水系建设工程；8、开工建设（10月底前）南湾开元文化旅游度假项目；9、开工建设沿大别山高速公路明港至鸡公山段；10、开工建设淮滨至内乡高速公路豫皖省界至淮滨段；11、开工建设G107绕市区段一级公路新建工程；12、开工建设袁湾水库；13、实施国家储备林基地建设项目；14、实施信阳产业新城项目；15、加快建设许昌至信阳高速公路信阳段；16、加快建设安阳至罗山高速公路上蔡至罗山段；17、持续推进平桥区中山铺项目；18、建成明阳新能源产业园；19、建成淮滨纺织服装产业园；20、建成淮滨至信阳高速息县至邢集段。

　　强化服务保障。实行项目台账式管理，坚持领导分包、重大项目周例会、联审联批会议、双月集中开工等制度，一个项目、一名领导、一套专班、一抓到底。持续推进土地利用综合改革，中心城区完成土地收储1万亩以上，出让5000亩以上；加快政府投融资平台市场化转型，完成信阳华信投资集团、鸡公山文旅集团、信阳发展投资公司、市水利投资公司、市宏信公司、市供销合作集团公司等所有市属国有企业资本投资运营公司改革；盘活行政事业单位存量资产，有效经营城市公共空间资源，探索城市公用设施资产证券化融资；大力争取土储债、公路债、棚改债等政府专项债券；依托多层次资本市场，加强金融机构协调配合，积极开展PPP工作，拓宽项目投融资渠道。

　　（三）推动工业扩量提质，增强经济发展支撑能力。实施“六大行动”，推动制造业高质量发展，实现规模以上工业企业增加值增长8.5%左右。

　　实施“重点企业提升”行动。对已筛选的10家高利税、高成长、引领型重点企业，制定“一企一策”专项提升方案，进一步提升企业竞争力。

　　实施“百企倍增”行动。对已筛选的103家发展前景好、市场需求大、技术含量高的企业，制定倍增方案，在资源、要素、资金上进行倾斜，推进企业实现销售收入和税收倍增。

　　实施“智能制造引领”行动。深入推进以智能制造为引领的“三大改造”，重点实施308个技改项目和智能化改造项目，集中精力建设一批智能车间；推动产业链上下游企业、供应链协同企业智能化改造，带动产业集群整体转型。

　　实施“传统产业提质”行动。立足禽肉、茶叶、山茶油、白酒等食品产业，以华英集团、羚锐集团、黄国粮业、文新茶叶、五谷春酒业、鸡公山酒业为主体，打造千亿级食品产业集群。加快建材产业产能整合和产品升级，深入推进珍珠岩、膨润土精深加工和高端应用，构建非金属新材料全产业链；助推信钢融入全省钢铁“23X”产业发展格局；推动现代家居产业发展壮大。引导“六县一区”纺织服装产业联动发展，培育千亿级纺织服装产业集群。扩大现有装备制造企业产品市场占有率，培育装备制造零部件专业化公司。

　　实施“新兴产业培育”行动。建立电子信息和新一代信息技术、新能源和新能源汽车、节能环保、大数据和超算、生命健康、军民融合产业重点项目和企业培育库，力争新兴产业产值增长15%以上。推动高新区智能终端和显示终端产业、罗山县电子元器件加工制造产业联动发展。打造明阳智慧新能源装备生产基地。支持开源环保发展水污染防治装备、土壤污染防治与修复技术装备，支持三元光电建设远红外应用产品研发和制造基地。建设灵山科技大数据存储示范基地和“云上大别山”大数据中心。加快建设军民融合产业园区。

　　实施“产业集聚区提质转型”行动。围绕主导产业和骨干企业，绘制产业链、价值链、服务链图谱，提升产业链发展水平。壮大明港钢铁加工和淮滨纺织服装加工贸易专业园区，打造上天梯绿色新材料特色园区，建设潢川现代食品园，培育平桥光学产业园、浉河金牛冷链物流和仓储物流产业园、潢开区大别山铁路货运综合物流园，开工建设林安（信阳）智慧物流园，推动南湾榕基产业园运营，建设高新区显示终端、风电装备、光电、新能源汽车、超算5个百亿级产业园和羊山新区家装产业园。选取2个产业集聚区作为市场化运营试点，由行政管理模式向市场化运营转变。加快潢川县和潢开区融合争创国家级经济技术开发区，确保信阳高新区创成国家级高新区。

　　（四）加强污染防治和生态建设，巩固提升绿色发展优势。深入贯彻习近平生态文明思想，保持加强生态文明建设的战略定力，让绿色成为信阳发展最靓底色。

　　坚决打赢大气污染防治攻坚战。持续加强机动车管控，开展城区“黑烟车”清零行动，6月底前规划区内柴油货车和非道路移动机械全部实现达标排放、所有渣土车实现规范化管理。加大工业排放治理，建立管控清单，实行错峰生产，6月底前完成中心城区282家重点涉气企业整改提标，9月底前实现中心城区所有工业企业在线监控联网运行。大力开展散煤清零专项行动，削减工业用煤量，全面拆改35蒸吨/时以下燃煤锅炉。强化扬尘治理，落实“六个百分之百”要求，力争中心城区道路积尘平均负荷下降到0.5克/平方米以下。加强餐饮油烟污染治理，本月底前完成中心城区所有餐馆检测整治、露天烧烤集中整治和洁净烧烤炉替代。今年是创建国家空气质量二类标准城市的关键一年，我们将强化过程管控，压实控尘、控车、控排三个专班责任，以壮士断腕的勇气、背水一战的决心，推进大气污染治理，为2020年创建成功奠定坚实基础。

　　大力推进“四水同治”。以“一横五纵三区”水资源均衡配置为总体布局，全面推进20项重点水利工程建设，打造“千湖之市、生态水城”。加快推进淮河重要支流系统治理、大中型病险水库除险加固、新建改建小型水库、史灌河治理、水系生态廊道造林绿化等6项在建工程，开工建设海营智慧城水系连通、浉河三期水环境综合治理、洪汝河（淮滨段）治理、白果冲水库建设等8项工程，完成东方河水库、龙王寺水库扩建、引九济石3项工程前期工作，力争出山店水库灌区、张湾水库、晏河水库3项储备工程前期工作取得较大突破。认真落实河湖长制，全力打好水污染防治攻坚战，推进黑臭水体治理示范城市建设，加快城区老旧供排水管网改造和污水再生利用设施建设，完成各县建成区黑臭水体截污纳管和排污口整治，实现第二、第三污水处理厂有效运行，确保中心城区和各县生活污水集中处理率分别达到95%和90%以上，中心城区黑臭水体基本消除，城市集中式饮用水水源水质达标率达到100%，全市地表水质量达到Ⅲ类水质以上，无劣V类水体，地下水质量考核点位水质级别保持稳定。

　　全面加强生态建设。大力实施国土绿化提速行动，巩固去冬今春新造林成果，启动绿色生态示范区建设。强力推进淮河廊道建设及生态修复，建立“人防+技防”“河长+警长”监管体系，打击非法采砂、规范合法采砂、修复河道生态，形成依法科学规范有序的河道采砂管理长效机制。综合整治露天矿山，加快上天梯矿山资源整合和绿色矿山建设。做好中心城区美化绿化，完成中心城区园林景观风貌总体规划设计和百花园片区、新五大道、新七大道、工五路、京港澳高速下线口、信阳东站进口绿化提升改造，中心城区绿化覆盖率提高到43%左右，成功创建国家森林城市，让全市人民享有更多青山碧水、蓝天白云！

　　（五）深化改革开放创新，激发发展动力活力。坚定不移推进改革开放再出发，让改革效力充分释放、开放之门越开越大、创新之势越聚越强。

　　深化重点领域改革。深化国有企业改革，妥善做好依法破产企业终结工作，9月底前全面完成“僵尸企业”职工安置任务；实现驻信央企、省属企业办社会职能全部剥离，完成市属国有企业“三供一业”分离移交。深化财税金融改革，全面实施预算绩效管理，稳妥划转非税收入征管职能。深入推进农村信用社改革。建立健全生态环保体制，试行生态环境损害赔偿制度。继续深化教育、科技、卫生健康、林业、粮食、盐业等领域改革。

　　持续扩大开放招商。主动融入“一带一路”及“三区一群”战略，推动信阳经济技术开发区、综合保税区、空港经济区建设，进一步提升开放平台优势和通道优势。坚持“高层次、小分队、精准性”招商方法，大员上前线，围绕产业链招商引资和招才引智，实现新增落地项目300个、总投资额不低于1000亿元、到位资金不低于300亿元。继续在“一核两翼”地区深耕细作，加强与武汉、合肥的经济联系，设立武汉、合肥招商办事处，鼓励发展“飞地经济”，合作共建产业园区。持续实施回归工程，充分发挥“驻外招商”“服务落地”“找老乡”三个专班作用，巩固信商大会成果，提高合同履约率、资金到位率和项目开工率。

　　深入实施创新驱动战略。持续抓好创新引领型企业、人才、平台、机构建设，大力实施“十百千”转型升级创新专项，加快创建国家创新型城市。突出企业创新主体地位，加强知识产权保护，培育省级创新主体10家左右。实施双创基地提质升级行动，引导行业骨干企业建设专业化众创空间，推动双创平台载体与互联网融合创新。积极争取“中原千人计划”，力争培育院士工作站3家。加快国家农业科技园区建设，力争获批省级科技创新平台7家以上，建设国家级质检中心1个、省级质检中心3个。扎实推进“四主体一联合”新型研发平台建设。设立科技金融引导风险补偿资金池，试行科技创新券制度，加大科技创新政策扶持。

　　大力优化营商环境。持续深化“放管服”改革，全面推行代办制、联审联批、并联审批、容缺办理，大力推广多评合一、企业投资项目承诺制等模式，一般性企业投资项目和工程建设项目审批时限压减至100个工作日以内。全面开展营商环境社会评价，把评价权力交给服务对象，让服务对象评价政府部门；实施排名制度、公开制度、奖惩制度，将评价结果与年终绩效奖金挂钩、与评先奖优挂钩、与干部考核挂钩，“刀刃向内”推动政府职能转变。加强社会信用体系建设，政府带头践信守诺，新官要理旧账，政府作出的承诺必须坚决兑现、作出的部署必须落实到位。

　　支持发展民营经济。全面落实更大规模减税降费政策，消除民企在准入许可、经营运行、招投标等方面的壁垒。发挥政府采购引导作用，支持首台（套）产品应用推广。着力破解民营企业融资难题，推进产业发展基金落地，引导金融机构扩大信贷规模，确保新增贷款向民营企业、中小微企业倾斜。持续开展“帮促”活动，落实领导帮扶机制，切实为企业排忧解难。引导企业建立现代企业制度，大力弘扬企业家精神，在全社会营造亲商爱商敬商的浓厚氛围。

　　（六）增加公共服务供给，不断提高老区人民幸福指数。坚持决策以民生为首要、支出以民生为首位，让老区人民共享改革发展成果。

　　扎实办好民生实事。今年，集中力量再办好10件实事：1、完成中心城区37条老旧道路提质改造；2、打通中心城区4条关键卡口道路；3、新建扩建12所中小学校；4、新建改建中心城区二类以上公厕27座以上、每个县5座以上，开放一批沿街单位公厕；5、改造农村敬老院51个；6、新建改建10所社区卫生服务中心，推动村卫生室产权公有化，开展新生儿耳聋基因筛查项目标本检验；7、新建中心城区25个、每个县2—3个社区公园（街头游园），绿化中心城区山体和空闲地，全面消除黄土裸露现象，每个县打造1条绿化亮化美化示范街；8、购置新能源公交车100辆，增开公交线路5条，新建公交场站5处，完成公交体制改革，在全市普及一元公交；9、改造一批农贸市场、新建一批副食品超市；10、改造中心城区老旧小区14个，规范1/3老旧小区物业管理。

　　持续提升城市品质。以全国文明城市创建为统领，将百城建设提质工程向各县区推开，用城市建设高质量让群众生活更美好。完成信阳市总体城市设计和中心城区11个片区控规的评审报批，完成浉河区、平桥区控规编制和城市设计，推动城市“双修”和有机更新，加快固始县、光山县城市总体设计和所有县主要专项规划编制。支持潢川县撤县设市，加快建设副中心城市；支持固始县建设豫皖区域性中心城市。提升城市形象，完成火车站北广场区域征迁改造、南站房改造、南广场基础工程和新二十四大街改建一期路面工程；畅通城市通道，完成四一路铁路立交桥改扩建和跨浉河公园步行桥、南湾二号桥、新十八大街南段贯通基础工程、新七大道（南湾段）延长段建设，新五大道下穿铁路立交桥工程进度达到50%以上；完善城市功能，建成羊山公交中心枢纽站、高新区城东停保站和羊山森林植物园，完成信阳东站换乘中心、羊山公园主体工程和中心城区集中供热一期工程，加快建设“一校三馆”、完成年度投资1.4亿元，开工建设羊山体育公园，广泛开展全民健身，全力打造城区15分钟全民健身圈。坚持为民创建、创建为民，重点做好生态环境治“污”、交通环境治“堵”、市容卫生治“脏”、公共服务治“差”。狠抓城市网格化、精细化管理，完成市级数字城管平台升级，推进市综合执法监督管理平台建设，加快打造智慧城市。

　　健全社会保障体系。完善落实就业创业政策，持续实施全民技能振兴工程，抓好高校毕业生、退役军人等重点群体就业，鼓励支持外出务工人员返乡下乡创业，实现城镇失业人员再就业2.7万人，新增农村劳动力转移就业4.5万人。大力实施全民参保计划，继续提高城乡居民基础养老金最低标准、调增企业和机关事业单位退休人员基本养老金。实施80岁以上老人高龄津贴制度。进一步提升城乡低保、特困供养、孤儿养育等保障标准，加强残疾人权益保障。加大住房保障力度，新开工棚改安置房2.3万套，更好解决群众住房问题。

　　协调推进社会事业。优先发展教育，新建、改扩建幼儿园50所；持续推进城镇扩容、乡村小规模学校和乡镇寄宿制学校建设，着力消除“大班额”，确保浉河区、平桥区、固始县通过全国义务教育发展基本均衡县（区）验收；加快普及高中阶段教育；推进职业教育产教融合、校企合作；加快信阳师范学院、信阳学院新校区和信阳农林学院二期建设，全力支持信阳师范学院创建信阳师范大学，推进信阳航空职业学院与国内知名院校联合办学。加快建设“健康信阳”，大力推进县域紧密型医共体建设，持续加大基层卫生基础设施和人才建设力度，支持中医药事业发展。拓展学生饮用奶计划实施范围，积极开展牛奶进校园行动。完善现代公共文化服务体系，建设信阳美术馆、科技馆，实现基层综合性文化服务中心全覆盖。提升文化遗产保护利用水平，深入挖掘本土文化，叫响“豫风楚韵”文化品牌。

　　加强和创新社会治理。深化平安信阳建设，完成“雪亮工程”建设任务，推进“一村（格）一警”和村（居）法律顾问工作，纵深推进扫黑除恶专项斗争。加大地方金融监管力度，坚决守住不发生区域性系统性风险的底线。压实安全生产责任，健全应急管理体系，确保实现“一杜绝三下降”目标。启动国家食品安全示范城市创建。走好网上群众路线，进一步提升网络综合治理能力。学习新时代“枫桥经验”，落实信访工作责任制，加快“12345”政务服务热线整合升级。开展房地产领域问题楼盘信访突出问题化解攻坚行动，9月底前完成省定化解任务，集中精力解决企业土地办证等历史遗留问题。

　　加强国防教育、国防动员和国防后备力量建设，支持国防和军队改革，支持驻信解放军、武警部队、预备役部队建设，抓好民兵和人民防空建设，深入开展双拥共建，加强退役军人服务保障体系建设。全面贯彻民族政策，做好外事侨务、对台工作。支持工会、共青团、妇联等人民团体更好发挥桥梁纽带作用。加强统计、档案、气象、地方志、红十字、社科研究、防震减灾、机关事务管理、援疆等工作。

　　三、2019年政府自身建设

　　面对发展新形势、人民新期待，政府工作要有新气象、新作为。我们将以“干部作风提升年”活动为抓手，努力打造勤政务实、廉洁高效、人民满意的政府。

　　（一）坚定政治站位。深入学习习近平新时代中国特色社会主义思想，树牢“四个意识”，坚定“四个自信”，坚决做到“两个维护”，始终在思想上政治上行动上同以习近平同志为核心的党中央保持高度一致。加强政府系统党的政治建设，把党的要求贯彻到政府工作全过程、各领域，始终做到坚决贯彻不走样、忠实执行不打折、狠抓落实不懈怠。扎实开展“不忘初心、牢记使命”主题教育，牢牢把握守初心、担使命，找差距、抓落实的总要求，汇聚推进“五个信阳”建设的强大合力。

　　（二）加强法治建设。深入贯彻全面依法治国基本方略，坚持依法行政，弘扬法治精神，加快建设法治政府。完善依法决策、科学决策、民主决策机制，自觉接受人大法律监督、工作监督和政协民主监督，主动接受社会监督和舆论监督，认真听取各民主党派、工商联、无党派人士和各人民团体的意见，进一步提高人大代表建议和政协委员提案办理水平。全面推进政务公开，管好用好各级政府网站，打造政府信息公开第一平台，让权力在阳光下运行，确保政府所干都是人民所盼。

　　（三）勇于担当作为。坚持斗争精神，提升斗争本领，砥砺斗争意志，不回避矛盾，不推卸责任，迎难而上，敢啃硬骨头，确保政府工作干一件成一件。弘扬焦裕禄“三股劲”精神，加强政策学习研究，加强公务人员培训，践行“一线工作法”，全面锤炼“五个过硬”、增强“八种本领”。将重大决策部署、重点工作任务、重点工程项目列入“干部作风提升年”联合重点督查内容，综合运用政务督查、现场观摩、电视问政、群众评议、媒体曝光等手段推动落实。建立双向激励约束机制，旗帜鲜明为敢于担责担难担险干部撑腰鼓劲，严厉整肃庸政懒政怠政行为，让愿干事、敢干事、干成事成为广大干部的自觉追求。

　　（四）坚持廉洁从政。认真履行全面从严治党主体责任和“一岗双责”，切实做好巡视整改“后半篇文章”。牢固树立过紧日子思想，一般性支出压减5%以上，“三公”经费只减不增，把有限的资源和财力用在降负惠企、让利裕民上。严格执行中央八项规定精神，大力倡树“五比五不比”，坚决反对和整治一切形式主义、官僚主义，切实减轻基层负担，让干部从文山会海、迎评迎检、材料报表中解脱出来，集中精力干事创业。全力支持纪委监委和审计监督工作，深入抓好“以案促改”，严惩重点工程和重点领域腐败问题，保证干部清正、政府清廉、政治清明。

　　各位代表！奋进新时代，逐梦新征程，只有干出来的精彩，没有等出来的辉煌。让我们更加紧密地团结在以习近平同志为核心的党中央周围，高举习近平新时代中国特色社会主义思想伟大旗帜，在省委省政府和市委的坚强领导下，同心共筑中国梦、争先进位谋出彩，以优异成绩庆祝中华人民共和国成立70周年!
